# Anaerobic Energy Expenditure and Mechanical Efficiency during Exhaustive Leg Press Exercise

**DOI:** 10.1371/journal.pone.0013486

**Published:** 2010-10-19

**Authors:** Esteban M. Gorostiaga, Ion Navarro-Amézqueta, Roser Cusso, Ylva Hellsten, Jose A. L. Calbet, Mario Guerrero, Cristina Granados, Miriam González-Izal, Javier Ibáñez, Mikel Izquierdo

**Affiliations:** 1 Studies, Research and Sport Medicine Center, Government of Navarre, Navarre, Spain; 2 Department of Physiological Sciences I, Institut d'Investigacions Biomèdiques August Pi i Sunyer (IDIBAPS), University of Barcelona, Barcelona, Spain; 3 Copenhagen Muscle Research Centre, Molecular Physiology Group, Section of Human Physiology, Department of Exercise and Sport Sciences, University of Copenhagen, Copenhagen, Denmark; 4 Department of Physical Education, University of Las Palmas de Gran Canaria, Las Palmas de Gran Canaria, Spain; Pennington Biomedical Research Center, United States of America

## Abstract

Information about anaerobic energy production and mechanical efficiency that occurs over time during short-lasting maximal exercise is scarce and controversial. Bilateral leg press is an interesting muscle contraction model to estimate anaerobic energy production and mechanical efficiency during maximal exercise because it largely differs from the models used until now. This study examined the changes in muscle metabolite concentration and power output production during the first and the second half of a set of 10 repetitions to failure (10RM) of bilateral leg press exercise. On two separate days, muscle biopsies were obtained from vastus lateralis prior and immediately after a set of 5 or a set of 10 repetitions. During the second set of 5 repetitions, mean power production decreased by 19% and the average ATP utilisation accounted for by phosphagen decreased from 54% to 19%, whereas ATP utilisation from anaerobic glycolysis increased from 46 to 81%. Changes in contraction time and power output were correlated to the changes in muscle Phosphocreatine (PCr; r = −0.76; P<0.01) and lactate (r = −0.91; P<0.01), respectively, and were accompanied by parallel decreases (P<0.01-0.05) in muscle energy charge (0.6%), muscle ATP/ADP (8%) and ATP/AMP (19%) ratios, as well as by increases in ADP content (7%). The estimated average rate of ATP utilisation from anaerobic sources during the final 5 repetitions fell to 83% whereas total anaerobic ATP production increased by 9% due to a 30% longer average duration of exercise (18.4±4.0 vs 14.2±2.1 s). These data indicate that during a set of 10RM of bilateral leg press exercise there is a decrease in power output which is associated with a decrease in the contribution of PCr and/or an increase in muscle lactate. The higher energy cost per repetition during the second 5 repetitions is suggestive of decreased mechanical efficiency.

## Introduction

Whereas the physiological and metabolic responses to maximal running, cycling and knee extension exercise using the leg extension ergometer have been well documented, to our knowledge, careful investigations examining substrate utilisation across a single set of bilateral leg press exercise have not been performed. Bilateral leg press exercise is a multi-joint (hip, knee, and ankle) exercise and is one of the most common resistance training type of exercise used to enhance performance in sports and in knee rehabilitation as it produces greatest neural activation than the majority of weight-bearing knee extension exercises [1].

Information about anaerobic energy production and mechanical efficiency (expressed as external work or power done per a given ATP produced) [2], [3] that occurs over time during short-lasting maximal exercise is scarce and controversial [4]. A limited number of studies that employ cycle exercise [5], [6], static knee-extension [7]–[9] or dynamic one-legged knee-extensor [4], [10]–[12] exercise at a constant work rate [10] have estimated that ATP utilisation per work unit either decreased [7]–[10] or increased over time [4] during exercise. This may be partly due to the variety of experimental conditions, the mode of exercise chosen to estimate mechanical efficiency and the difficulties of quantifying anaerobic energy production based on the decrease in muscle adenosine triphosphate and PCr, as well as the accumulation of metabolites such as lactate.

Bilateral leg press is an interesting muscle contraction model to estimate anaerobic energy production and mechanical efficiency during maximal exercise because it largely differs from the models used until now. Thus, during a set of 10 repetitions of bilateral leg press exercise the duration of the muscle contraction is between 1500 to 2000 ms, the contraction frequency is ∼0.30 Hz, force and intramuscular pressures are high, the blood flow to the exercising muscle and the lactate released from muscle to the blood stream is highly restricted, power output declines over time and the external mechanical work produced during each muscle contraction remains constant. This leg press exercise model is different from the model of maximal cycling or treadmill running (contraction duration of ∼80–200 ms; contraction frequency of ∼2 Hz; lower force and intramuscular pressures, low blood flow restriction, and power output and external mechanical work declining with time) or the classical intermediate constant-load one-legged knee extensor model [4] in which the contraction duration is ∼500 ms, the contraction frequency is ∼1 Hz and power output and external mechanical work does not change during exercise. It is therefore still uncertain whether mechanical efficiency changes over time during short-lasting maximal dynamic exercise such as the leg press model.

The purposes of the present study were: 1) to examine changes in muscle metabolism and power output during the initial and final 5 repetitions of a set of 10 repetitions to failure of bilateral leg press exercise, 2) to identify the metabolic factors that could contribute to the decline in muscle power production during leg press exercise, and 3) to estimate the changes over time in anaerobic energy production and mechanical efficiency. One hypothesis was that the decline in power production during exercise would be related to changes in muscle metabolites. It was also hypothesised that changes in mechanical efficiency would occur over time during a set of exhaustive leg press exercise.

## Materials and Methods

### Subjects

Six healthy male volunteers participated in this study. Their mean (± SD) age, height, body mass, estimated maximal oxygen uptake (VO_2max_) in cycloergometer and maximal strength (1RM) during leg press exercise were 34±6 years, 179±5 cm, 74.5±7.2 kg, 57.1±4.9 ml·kg^−1^·min^−1^ and 199±43 kg, respectively. All were recreational athletes, mainly in endurance events, but none trained for competition. The subjects were thoroughly informed of the purpose, nature, practical details and possible risks associated with the experiment, as well as the right to terminate participation at will, before they gave their voluntary written consent to participate. A medical examination was also completed by a physician. The present study is part of a project which has been approved by the Institutional Review Committee of the Instituto Navarro del Deporte, according to the Declaration of Helsinki.

### Experimental design

This study was designed to examine metabolic responses elicited during one set of 10 repetitions to failure (10RM) of bilateral leg press exercise. Failure was indicated by the inability to complete the next repetition after 10 repetitions. Each subject participated in two experiments performed on separate days. In one experimental day the subjects performed a set of 5 repetitions of leg press exercise and on the other day a set of 10 repetitions were performed. Both experiments were performed with the same absolute load and averaged 168±39 Kg (∼85% of the individual 1RM). On both days percutaneous muscle biopsy samples were obtained from the vastus lateralis prior and immediately after the last repetition. All subjects participated in the two experiments in random order. The two randomly assigned main tests were performed at the same time of the day and were separated by one to two months. Subjects were requested to repeat their pre-recorded normal diet and refrain from any form of intense physical exercise for 48 h prior to each test.

### Preliminary tests

Several pre-test sessions were done during the 3 weeks preceding the experiments. First, the subjects were familiarised with the experimental testing procedures about 2 week beforehand. Second, two weeks before the first experiment subjects participated in a control testing day where resistance-load verifications for one repetition maximum strength (1RM: the heaviest load that could be correctly pressed only once using the correct technique) and for 10 repetition maximum strength (10RM: the individual maximum load that produced 10 repetitions to fatigue) were determined in the leg press exercise machine. After measuring 1RM, we estimated the absolute load that should produce 10 repetitions to fatigue (10RM). Then, after at least 10 min rest, the subjects performed a maximal repetitive set until failure with the load that theoretically should produce 10 repetitions to fatigue (∼85% of 1RM). If the number of repetitions until failure was equal to 10, the load was defined as a 10RM and used during the experimental main tests. If the number of repetitions until failure was different from 10, several trials of a maximal repetitive set until failure were performed on different days with lower or higher loads during subsequent test sessions, in order to determine the load leading to failure in exactly 10 repetitions. Third, on a separate day the maximum oxygen uptake (VO_2max_) of each subject was estimated [13] by using a continuous incremental test until exhaustion on a friction-loaded cycle ergometer (Monark Ergomedic 818E, Varberg, Sweden). The first work load (60 W) was high enough to ensure that exhaustion would occur within 8–14 min, the load being increased by 30 W at the end of every min. Heart rate was monitored throughout the test continuously (15 s) with a cardiotachometer (Sportester Polar, Kempele, Finland). Average power output at exhaustion was 347±27 W.

### Main tests

On the morning of the experiment, the subject arrived after a light breakfast and 2 h fast period. On arrival at the laboratory subjects rested on bed for 20 minutes in order that small incisions could be made under local anaesthesia (1% lidocaine) through the skin and fascia over the vastus lateralis muscle of one leg. The subjects then completed a period of warm-up consisting of a set of 5 repetitions at 50%, three to four repetitions at 75% and 1 repetition at 90% of maximal bilateral leg press strength (1RM). Three to four subsequent attempts were made to determine the 1RM. The resting period between maximal attempts was always 2 min. After 10 min rest, a muscle biopsy (resting biopsy) was taken from muscle vastus lateralis and arterialised blood sample was drawn from the earlobe previously hyperemized with a warming ointment (Finalgon, Boehringer Ingelheim, Germany). Then an intense bilateral leg press exercise, 5 or 10 repetitions with the maximum load possible to achieve 10 repetitions (10RM), was performed. In each repetition the subject was instructed to move the weight as fast as possible. The time for each repetition and the concentric and eccentric components were recorded. There was about 1-s pause between repetitions to ensure that the stretch-shortening cycle did not enhance performance of the subsequent concentric action. The power output of each repetition was monitored continuously and measured during the concentric phase of leg press action. Immediately after the last repetition (within 5–10 s) an additional muscle biopsy and arterialised blood samples were taken. All subjects were highly motivated and strong verbal encouragement was given to all subjects to motivate them to perform each repetition maximally and as rapidly as possible.

### Equipment

The study was performed on a horizontal bilateral leg extension variable resistance machine (i.e. leg press action in a sitting position) (Technogym, Gambettola, Italy). The sitting was individually adjusted to minimise displacement between the lower back and backrest during muscular force exertion and, therefore, to avoid posture change. Each subject was instructed to fix their feet in the same position on the force platform to ensure a constant foot position. The exercise machine incorporated four force transducers on a foot platform located below the subject's feet. The strain gauges recorded the applied force (N) within an accuracy of 1 Newton at 1000 Hz. The force platform and leg press plate all remained stationary throughout the lift, while the body moved away from the feet. In addition, a rotational encoder (Computer Optical Products Inc, California, USA) was attached to the weight plates to record the position and direction of the displacement within an accuracy of 0.2 mm at 1000 Hz. Customised software was used to calculate power (immediate product of displacement velocity and applied force) and work output (vertical displacement of the weight plates multiplied by applied force) per repetition. After the end of the exercise, results were integrated over 1-ms intervals. The maximum 10-ms integral of applied force and displacement velocity during each repetition is referred to as “peak power output”. The average 10-ms integral of applied force and displacement velocity over the total concentric contraction time of each repetition is referred to as “mean power output”.

### Muscle samples

Muscle biopsies, as described by Bergstrom [14], were taken from the vastus lateralis muscle of the left leg before and immediately after 5 and 10 repetitions of the leg press exercise. The muscle sample was immediately frozen (in 5–10 s) in liquid nitrogen and stored at −80°C for subsequent metabolite assay, after being freed from visible fat and connective tissue.

### Analysis

Muscle Phosphocreatine (PCr), creatine (Cr), lactate, glucose 1-phosphate (G-1-P), glucose 6-phosphate (G-6-P), fructose 6-phosphate (F-6-P) and free glucose were analysed by fluorometic analysis [15]. Skeletal muscle adenine nucleotides and inosine monophosphate (IMP) were analysed by high-performance liquid chromatography (HPLC) using a modified version [16] of the method originally described by Dellevold et al. [17]. All muscle metabolite concentrations are expressed as mmol·Kg^−1^ wet muscle.

### Calculations

Total average ATP production (mmol·Kg^−1^ wet muscle) from anaerobic sources, assuming a closed system, was estimated as previously described by Chasiotis et al. [2], from changes in average metabolite values obtained before and immediately after exercise using the following formula:
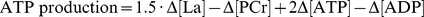
where Δ refers to exercise-induced change in each metabolite. The relative contributions to ATP utilisation from ATP and PCr degradation were calculated from concentration changes during the period, whereas the amount of ATP re-synthesised through glycolysis was calculated from the lactate formed, assuming 1.5 mmol ATP·mmol^−1^. Although it is recognized that some lactate may have left the muscle during exercise, this calculation ignores lactate released to the blood during exercise and assumes that blood flow in the active muscles is arrested during the ∼30 s of the exercise [6], [18], [19] and that the majority of carbohydrate substrate originates from muscle glycogen. This creates a complete anaerobic situation [20]. It also assumes that decreases in muscle ATP concentration are accounted for by increases in inosine 5′-monophosphate (IMP).

The average anaerobic ATP utilisation rate (mmol·Kg^−1^ wet muscle·s^−1^) was obtained by dividing the average anaerobic ATP production by the total duration of the exercise.

The minimum average anaerobic glycogenolytic and glycolytic rates during each experiment were calculated from accumulation of glycolytic metabolites using the formulas described by Hultman and Sjoholm [20], as follows:
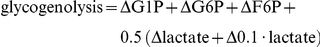


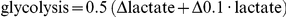



The values were divided by time to give millimole glycosyl units per kilogram per second (mmol glucosyl · units Kg^−1^ wet muscle·s^−1^). Increases in intracellular glucose have not been included in the calculation of glycogenolysis because the free glucose in muscle was probably released by the action of the debranching enzyme and not the action of phosphorylase [21].

The concentration of inorganic phosphate (P_i_) in the muscle after each exercise was calculated from changes in ATP, ADP, PCr and hexose monophosphates (HMP) using the formula [2]:
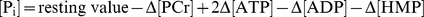
A resting value of 2.9 mmol·l^−1^ was used [2].

Muscle pH was estimated from muscle lactate content by using the Sahlin et al. [22] equation.

Cellular energy charge, a measure of the extent to which the total adenine nucleotide pool of the cell (ATP, ADP and AMP) is phosphorylated, was estimated by using the following equation [23]:
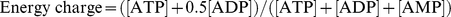



### Blood samples

Capillary blood lactate and ammonia samples were obtained from a hyperemic earlobe. After cleaning and puncturing, a 5-µl sample of whole blood was automatically aspirated into a single use enzyme-coated electrode test strip. Lactate concentration was determined via amperometric measurement and the result displayed in 60 s (Lactate Pro LT-1710; Arkray KDK Corporation, Shiga, Japan). In terms of reliability, the manufacturers report a coefficient of variation (CV) of 3.2% and 2.6% with lactate standards of 2 and 11 mmol·l^−1^, respectively. The Lactate Pro was checked for accuracy according to manufacturer's instructions before every test. A single 20 µl of whole blood sample was also taken from hyperemic earlobe with an Eppendorf varipipette and immediately analysed for ammonia concentration with an ammonia checker (BAC) II (model AA-4120, Kyoto Daiichi, Kayaku Co., Ltd. Japan, Menarini Diagnostic, Italy). This analyser uses a reflectometer to optically measure the reflection intensity (45°) of reagent colour reaction in biochromatic mode and was calibrated before and after every test with a known control (58.7 µmol·l^−1^).

### Statistical analysis

Standard statistical methods were used for the calculation of mean and standard deviation (SD). Student's paired t-test was used for comparisons of analytic values during the two different experimental conditions in this study, whereas one-way analysis of variance for repeated measures was used to examine the differences in performance indexes and metabolite concentrations over time. When a significant F-value was achieved (P<0.05), the means were compared using a LSD post-hoc test. For the purposes of comparison the power output for the second 5 repetitions were compared with the first 5 repetitions. Pearson product-moment correlation test was performed to examine the relationship between variables. Statistical significance was accepted at the P<0.05 level.

## Results

### Power and force production

**Figure 1:**
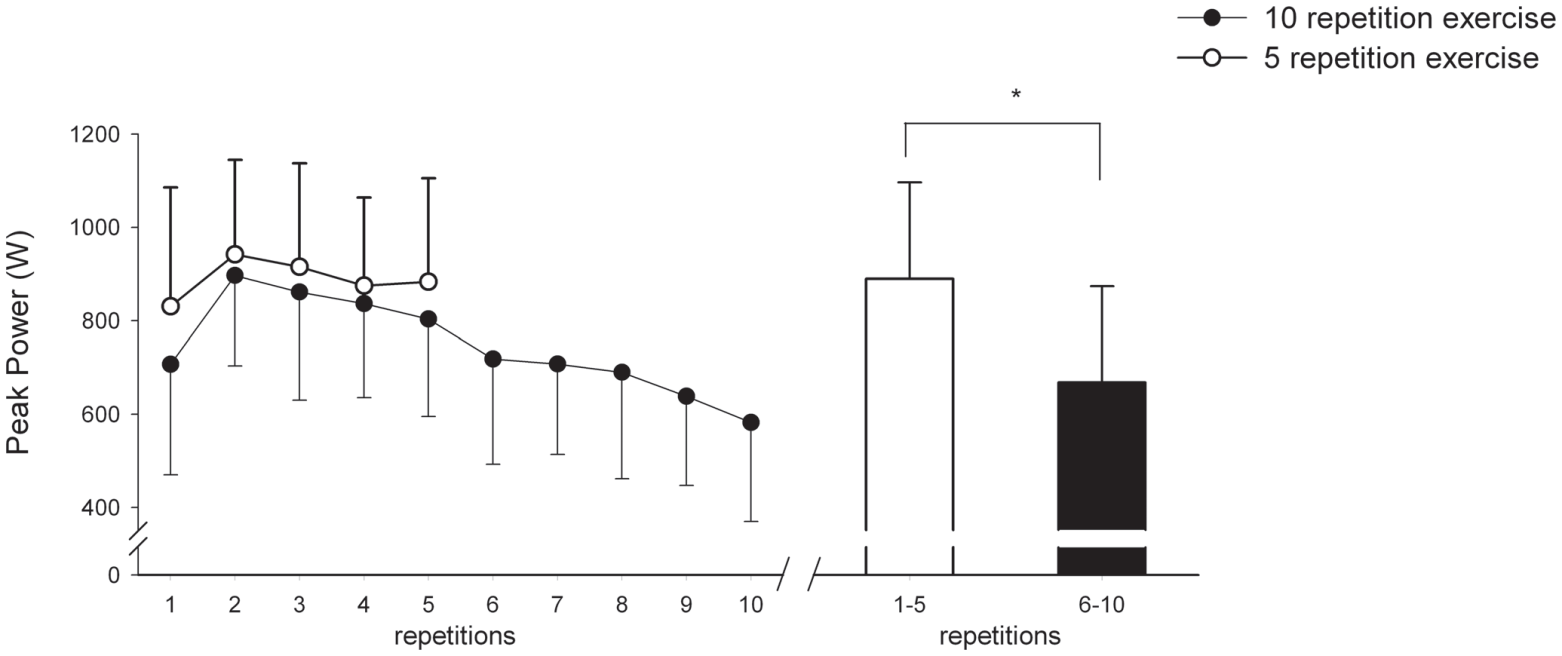
Peak power output profiles (average for n = 6 subjects) for each exercise during the two experimental conditions: when exercise was 5 repetitions (open circles) and when exercise was 10 repetitions (filled circles). Boxes represent mean of the peak power output for the first and the second half of a set of 10 repetitions. *significant difference (P<0.01) between the first and the second 5 repetitions. Values are means ± SD.

The peak power output profiles during each repetition in the two experimental conditions (5 or 10 repetitions) are shown in Figure 1. Average peak power production during the first 5 repetitions was similar in both experimental periods. The highest average peak power for a leg extension was recorded within the 2-3 initial repetitions of the exercise and amounted to 897±194 W. The average peak power was well maintained during the first 5 repetitions of the exercise, however after that peak power declined progressively during exercise (P<0.05), reaching a value of 64±17% of the highest value after 10 repetitions. Average peak power decreased 19±12% (P<0.05) from the first (821±207 W) to the second (667±206 W; Fig. 1) 5 repetitions of the exercise. The magnitude of the decline in average mean power production between the first and the second 5 repetitions was 23±10% (from 349±68 to 268±58 W: P<0.05).

**Figure 2:**
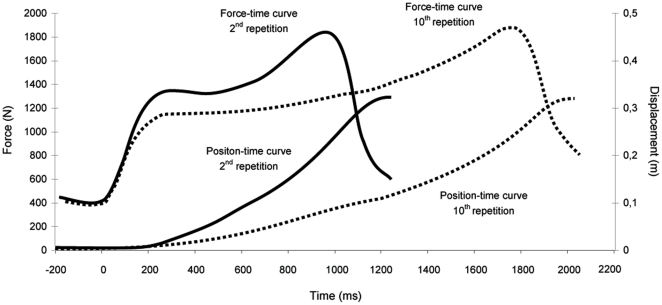
Curves of the position-time of the encoder attached to the weight plates, and applied force-time on the force platform by one representative subject, during the second (solid line) and the tenth (dash line) repetition of a set of 10 repetitions. This profile was similar for all subjects.

The total duration of exercise was 32.6±4.4 s: i.e. 14.2±2.1 s for the first 5 repetitions and 18.4±4.0 s for the second 5 repetitions (P<0.05). During the first 5 repetitions average contraction time per repetition was 1.59±0.3 s and average relaxation time per repetition was 1.25±0.26 s, whereas during the second 5 repetitions the corresponding contraction and relaxation times were 20–25% longer (1.96±0.35 s and 1.71±0.56 s, respectively) compared with the first 5 (Fig. 2). For each second of exercise the contraction/relaxation cycle was similar for the first and the second 5 repetitions (∼0.55). The total amount of work performed was slightly (3%) but significantly less (P<0.05) during the second 5 repetitions (433±92 J) than for the first 5 repetitions (453±87 J). This drop was due to a slight decline (P<0.05) in the load displacement from 35.5±5.6 cm for the first 5 repetitions to 34.3±6.3 cm for the second 5 repetitions.

### Muscle metabolites

Tables 1 to [Table pone-0013486-t002] 3 show muscle metabolite concentrations at rest and after exercise. Resting metabolite concentrations, averaged from both experimental days, were within the normal range for human skeletal muscle. Concentrations of ATP, AMP and IMP were unchanged during exercise (Table 1). The ADP concentration increased (P<0.05) during the first 5 repetitions, and remained high at the end of exercise. PCr concentration decreased to 58% and 38% of the resting value after the first and the second 5 repetitions, respectively. Changes in calculated P_i_ generally followed those of PCr. Compared with rest values the energy charge decreased (P<0.05) after 5 and 10 repetitions. The calculated ATP/ADP ratios decreased by 9% (P<0.05) after 5 repetitions and remained at this level until the end of the exercise (Table 2). The ATP/AMP ratio did not change after 5 repetitions but decreased by 19% (P<0.05) at the end of the exercise. No change occurred in either the ADP/AMP and ATP/IMP ratios throughout exercise.

**Table 1:** Effects of leg press exercise on adenine nucleotides, IMP, PCr, Cr, P_i_ and energy charge at rest and during exercise.

	Pre exercise			Mid exercise (5 reps)			Post exercise (10 reps)		
ATP	6.52	±	0.38	6.19	±	0.59	6.42	±	0.57
ADP	0.85	±	0.03	0.89	±	0.08 a	0.91	±	0.10
AMP	0.08	±	0.04	0.08	±	0.03	0.09	±	0.03
IMP	0.01	±	0.00	0.01	±	0.00	0.08	±	0.11
TAN	7.44	±	0.40	7.15	±	0.66	7.41	±	0.67
PCr	20.24	±	6.31	11.68	±	7.82	7.74	±	5.53 ^ab^
Cr	8.66	±	3.92	16.97	±	6.33	25.45	±	3.8 a
PCr + Cr	28.9	±	3.94	30.56	±	6.19	34. 55	±	6.23
Energy charge	0.933	±	0.01	0.927	±	0.01 a	0.927	±	0.004 a
P_i_		2.9		17.63	±	15.74	24.8	±	15.8 a

Values are means ± SD in mmol·kg^−1^ wet muscle, except [P_i_] (mmol·l^−1^ intracellular water) and energy charge; n = 6, except at post exercise, where n = 4-5. TAN, total adenine nucleotides (ATP + ADP + AMP); IMP, Inosine 5′-monophosphate; Cr, Creatine. For calculations of P_i_ and energy charge see METHODS.

a significant difference (P<0.05) with pre exercise value.

b significant difference (P<0.05) with middle exercise value

**Table 2 pone-0013486-t002:** Effects of leg press exercise on nucleotide metabolite ratios at rest and during exercise.

	Pre exercise			Middle exercise (5 reps)			Post exercise (10 reps)		
**ATP/ADP**	7,7	±	0,3	7,0	±	0,3 a	7,1	±	0,2 a
**ATP/AMP**	95,9	±	27,2	88,4	±	24,8	77,8	±	19,7 a b
**ADP/AMP**	12,4	±	3,4	12,6	±	3,3	10,9	±	2,7
**ATP/IMP**	1051.3	±	50.5	1055.3	±	23.2	648.4	±	553.8

Values are means ± SD.

a significant difference (P<0.05) with pre exercise value.

b significant difference (P<0.05) with middle exercise value.

**Table 3:** Effects of leg press exercise on glycolytic intermediates at rest and during exercise.

	Pre exercise			Middle exercise (5 reps)			Post exercise (10 reps)		
Glucose	0.47	±	0.17	0.52	±	0.38	0.37	±	0.27
G-1-P	0.01	±	0.01	0.03	±	0.03	0.06	±	0.05
G-6-P	0.07	±	0.05	0.62	±	0.74	1.43	±	1.05
F-6-P	0.02	±	0.01	0.08	±	0.07	0.09	±	0.05 ^a^
Lactate	1.86	±	0.95	7.1	±	2.54 ^a^	17.2	±	3.5 ^ab^
pH	7.02	±	0.02	6.93	±	0.05^ a^	6.79	±	0.14^ ab^

Values are means ± SD in mmol·kg^−1^ wet muscle; n = 6, except at post exercise, where n = 4-5. G-1-P, Glucose 1-Phosphate; G-6-P, Glucose 6-phosphate; F-6-P, Fructose 6-phosphate. For calculations of pH, see METHODS.

After the first 5 repetitions there was a 3.5-fold increase in the concentration of muscle lactate (P<0.05), whereas a more marked increase (∼ 9 fold) was noticed during the second 5 repetitions (P<0.05) (Table 3). The muscle concentration of F-6-P increased significantly (P<0.05) from rest up to the tenth repetition. In contrast, the muscle concentrations of free glucose, G-1-P and G-6-P did not increase above resting values during exercise. By using the Sahlin et al. [22] equation to relate changes in muscle lactate concentration to changes in average muscle pH it can be calculated that in the present study the first 5 repetitions would have resulted in a fall (P<0.05) in intramuscular pH from 7.02±0.02 (rest) to 6.93±0.05 and a further decrease (P<0.05) to 6.79±0.14 after the second 5 repetitions.

During exercise the energy was mainly derived from utilisation of high-energy phosphates and glycolysis. Table 4 shows the summary of measurements and estimates of ATP production from anaerobic sources during exercise made from the average values of the Tables 1 and 2. Assuming a closed system, the minimum anaerobic ATP production over the whole exercise (10 repetitions) calculated from changes in muscle lactate, phosphocreatine, and ATP concentrations was 35.7 mmol·Kg^−1^ wet muscle while glycogenolysis accounted for 64% and phosphagens contributed to 36% of the anaerobic ATP produced. When the whole exercise was divided into two parts, the minimum anaerobic ATP production was 9% higher during the second 5 repetitions (18.6 mmol·Kg^−1^ wet muscle) than during the first 5 (17.1 mmol·Kg^−1^ wet muscle). The fraction of ATP resynthesis derived from phosphagens decreased from the first 5 repetitions (54%) to the second 5 (19%) whereas the fraction of ATP production derived from glycogenolysis increased from the first 5 repetitions (46%) to the second 5 (81%).

**Table 4:** Summary of measurements and estimations of average ATP production from anaerobic metabolism.

	1 to 5 rep	6 to 10 rep	1 to 10 rep
Assuming a closed system			
Phosphagens	9.2	3.5	12.6
Glycogenolysis	7.9	15.1	23.0
ATP Turnover	17.1	18.6	35.7
Assuming an open system			
Estimated lactate release	4.5	7.1	11.6
ATP turnover	21.6	25.6	47.2

Mean ATP turnover estimations in mmol·kg^−1^ wet wt. Calculation for released lactate data is derived from product of measured increase in blood lactate and the estimated magnitude of extracellular water space (22.38 l; 28). Active mass (10 kg) was calculated using data of Essen et al. (19).

The previous estimate does not take into account the amount of lactate released from the muscle during the exercise. However, some lactate efflux from the active muscle occurred during each bout of exercise, since blood lactate concentrations immediately after the end of the fifth (2.4±0.6 mmol·l^−1^) and the tenth (4.5±1.0 mmol·l^−1^) repetition were elevated compared with resting values (1.0±0.1 mmol·l^−1^). Assuming that: 1) lactate was uniformly distributed in total extracellular water space (22.4 l: about 30% of the whole body mass) [24], 2) no lactate removal occurred from this pool during exercise, 3) the biopsy represents the entire mass of all the muscle groups involved during exercise [24], and 4) 10 Kg of muscle are involved in leg press exercise [25], it may be calculated that a lactate concentration of approximately 3.02 mmol·Kg^−1^ wet muscle (22.4 · (2.4-1.0) · 10^−1^) and 4.66 mmol·Kg^−1^ wet muscle (22.4 · (4.5-2.4) ·10^−1^) escaped the muscle during the first and last 5 repetitions, respectively. Therefore, a lactate concentration of 7.68 mmol·Kg^−1^ wet muscle (3.02 +4.66) escaped the muscle during the entire 10 repetitions. Since average muscle lactate concentration increased by 5.2 (first 5 repetitions) and 10.1 mmol·Kg^−1^ wet muscle (second 5 repetitions) during these periods, lactate efflux represented approximately 37% (first 5 repetitions), 32% (second 5 repetitions) and 33% (whole exercise) of the total lactate produced. These estimates of lactate released from muscle during the present exercise (∼30% of total lactate production) are consistent with those measured during one-legged, dynamic knee-extensor exercise [10] and during 80-s electrical stimulations of the quadriceps muscle [26]. Assuming that 1.5 mol of ATP are produced for every mol of lactate accumulated, the addition of the above estimate of lactate escaped from the muscle should give a total estimated anaerobic ATP production of 21.6 mmol·Kg^−1^ wet muscle during the first 5 repetitions, 19% higher anaerobic ATP production during the last 5 repetitions (25.6 mmol·Kg^−1^ wet muscle), and a total production of 47.2 mmol·Kg^−1^ wet muscle during the entire exercise (Table 4). In this case phosphagens contributed to 46%, 15% and 29% of the total estimated anaerobic ATP produced during the first 5, second 5 and 10 repetitions respectively, while the corresponding contribution from glycogenolysis accounted for 54%, 85% and 71% respectively.

The mean rate of ATP utilisation decreased during exercise because during the second 5 repetitions (1.40 mmol·kg^−1^·s^−1^) the values were 8% lower than during the first 5 repetitions (1.53 mmol·kg^−1^·s^−1^) (Table 5). The ATP utilisation per repetition in relation to units of mean power (µmol·watt^−1^·rep^−1^) and units of work (µmol·J^−1^·rep^−1^), which is a measure of the energy cost is also reported in Table 5. The ATP utilisation per repetition per unit of power was 35% higher during the second 5 repetitions than during the first 5. The ATP utilisation per repetition per unit of work was 21% higher in the second 5 repetitions than in the first 5.

**Table 5:** Average ATP turnover rate and average ATP utilisation per repetition in relation to unit of power and unit of work.

	1 to 5 rep	6 to 10 rep	1 to 10 rep
ATP turnover rate (mmol·kg^−1^·s^−1^)	1.5	1.4	1.4
ATP utilisation per power unit per repetition (µmol·watt^−1^·rep^−1^)	4.9	7.7	6.3
ATP utilisation per unit work per repetition (µmol·J^−1^·rep^−1^)	9.5	12.0	10.9

The minimum anaerobic glycogenolytic and glycolytic rates during leg press exercise were estimated. The results of these calculations show that during the first 5 repetitions the glycogenolytic rate exceeded the glycolytic rate by 25%. The glycogenolytic rate of 0.25 mmol·Kg^−1^ wet wt·s^−1^ calculated during the first 5 repetitions increased to 0.35 mmol·Kg^−1^ wet wt·s^−1^ during the second 5, however this 40% increase was less than the 50% increase in glycolytic rate (from 0.20 mmol·Kg^−1^ wet wt·s^−1^ to 0.30 mmol·Kg^−1^ wet wt·s^−1^). During the second 5 repetitions, however, the glycogenolytic rate still exceeded the glycolytic rate by 17%.

### Blood metabolite

Blood lactate increased (P<0.01) from 1.0±0.1 mmol·l^−1^ at rest to 2.4±0.6 mmol·l^−1^ and 4.4±1.0 mmol·l^−1^ after 5 repetitions and 10 repetitions, respectively. Average blood ammonia concentrations were not different from resting levels (19.6±6.6 µmol·l^−1^) immediately after 5 (21.4±7.4 µmol·l^−1^) and 10 repetitions (28.3±12.6 µmol·l^−1^).

### Correlations

**Figure 3:**
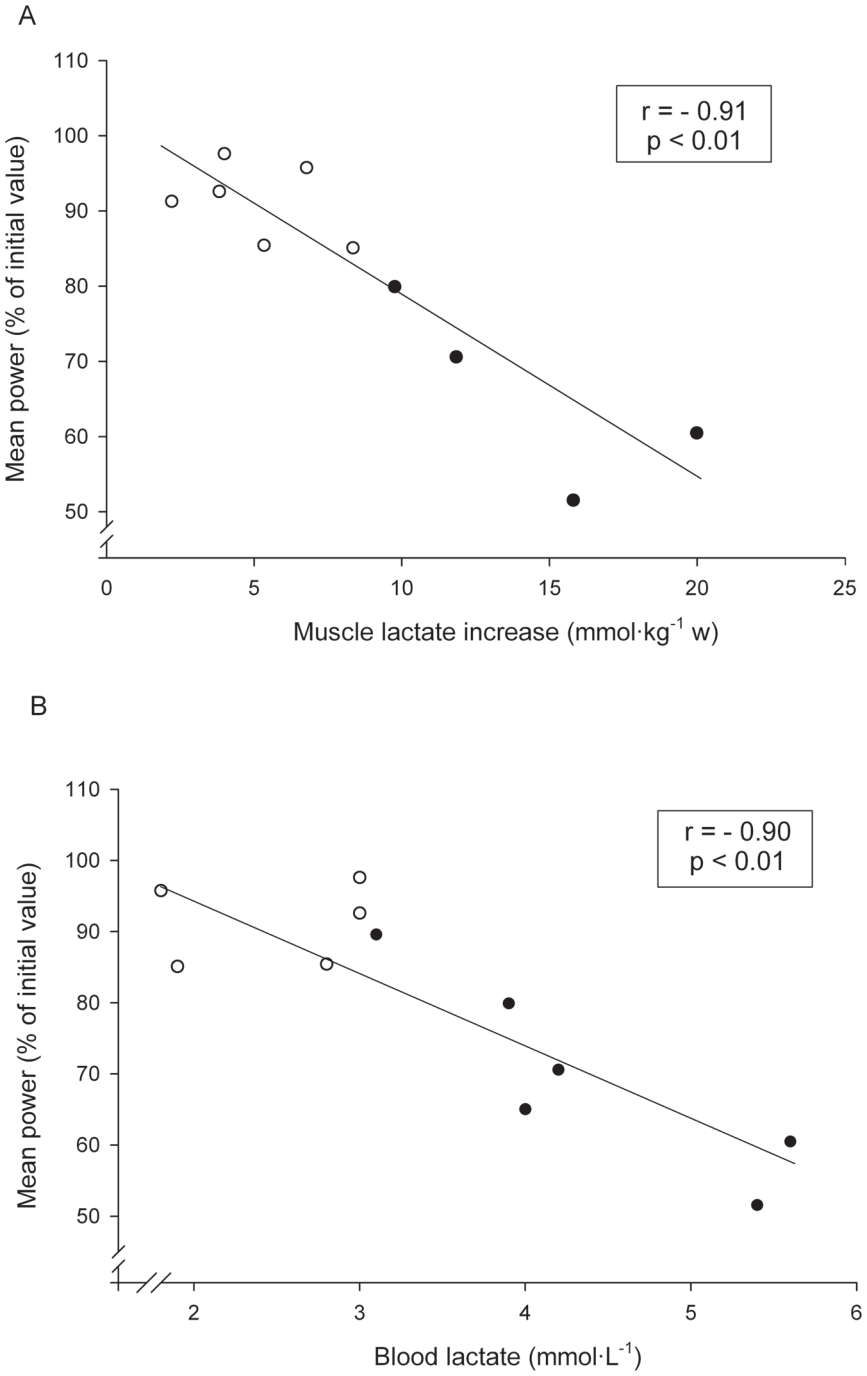
Relative mean power output changes and muscle lactate concentration increases. Individual relationships between the relative mean power output changes (expressed in percent of initial value) between the first and the last two repetitions of both experimental periods, and the muscle lactate concentration increases (3A) as well as with the final blood lactate concentration values (3B), during a set of 5 (open circles) and a set of 10 (filled circles) repetitions.

**Figure 4:**
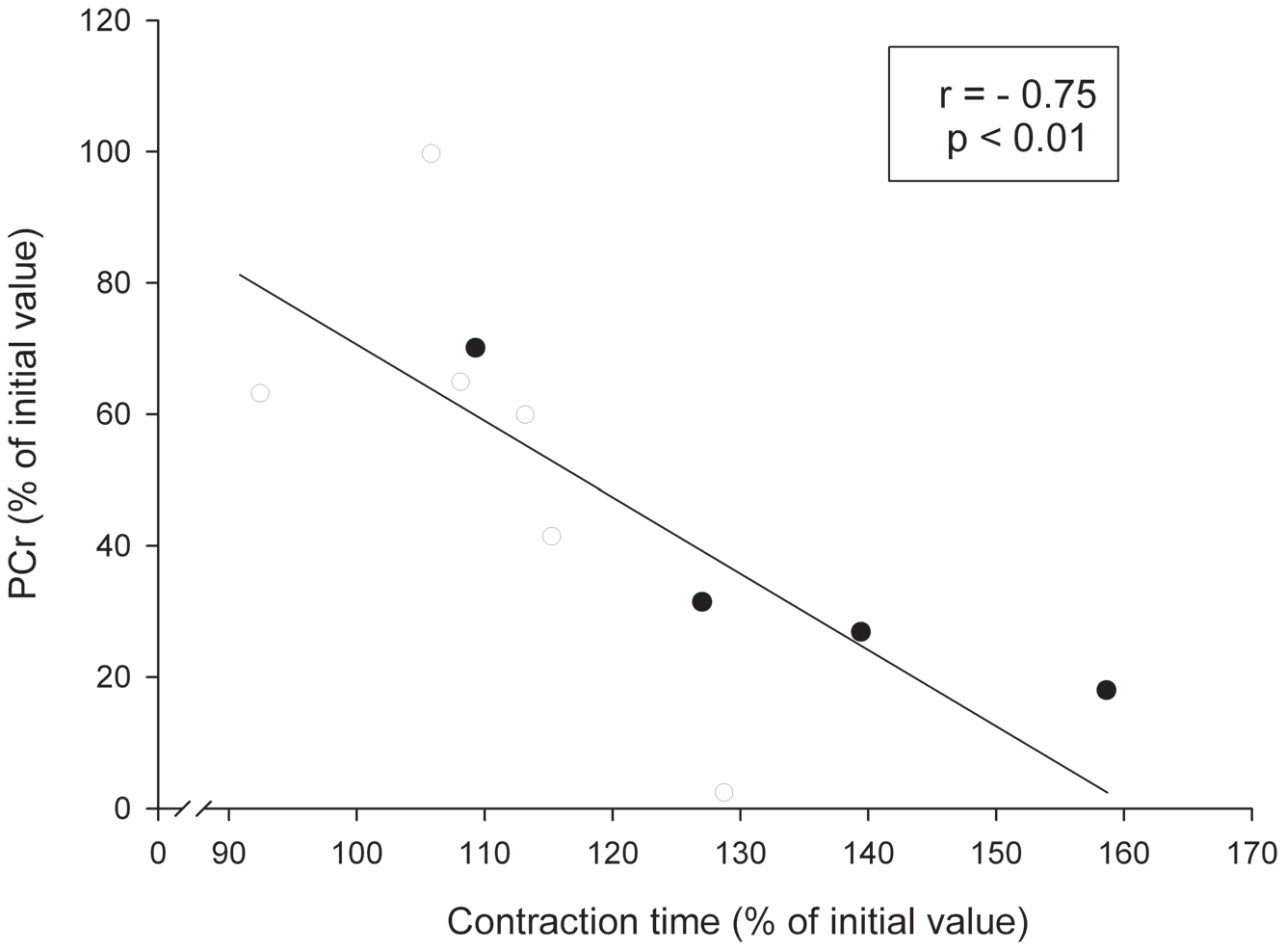
Individual relationship between PCr changes (expressed in percent of initial value) and the changes in the relative average duration of the concentric phase of leg press exercise (expressed in percent of initial value) during a set of 5 (open circles) and a set of 10 (filled circles) repetitions.

There was a negative correlation between individual contraction-induced relative changes (expressed as percent of initial values) in mean power output between the first (initial values) and the last (final values) two repetitions in both experimental periods, and individual changes in muscle (r = −0.91, P<0.01) (Fig. 3A) as well as blood (r = −0.90, P<0.01) (Fig. 3B) lactate concentrations. Similarly, inverse relationships (r = −0.75, P<0.01) were observed between the individual changes in PCr and the individual relative changes in the average duration of the concentric phase of leg press actions, expressed in percent of initial values (Fig. 4). A positive correlation (r = 0.89, P<0.01) was found between individual changes in muscle and blood lactate concentrations.

## Discussion

The primary aims of the present study were to determine the changes in muscle metabolite concentration and power output production during the first and the second 5 repetitions of a 10 repetition to failure of bilateral leg press exercise, and secondly to relate these changes to decreases in power. The main findings were: 1) the major reduction in PCr concentration mainly occurred within the first 5 repetitions of exercise, whereas muscle lactate accumulation was more substantial during the second 5 repetitions, and 2) a significant drop in power output was observed from the first to the second 5 repetitions, correlating with the reduction in phosphagens and the accumulation of muscle lactate, and accompanied by a parallel decrease in muscle energy charge as well as increases in ADP content. The present data shows that during the first 5 repetitions energy is primarily derived from PCr with a smaller contribution of glycolysis, while during the second 5 repetitions glycolysis was the major energy source. Finally, our study shows that the rate of ATP turnover per work unit increases as the exercise progresses, implying that mechanical efficiency is reduced during the second half of the exercise.

### Muscle metabolites

The bilateral leg press exercise produced similar fatigue profile and changes in muscle PCr, lactate and glycolytic intermediates over ∼35 seconds as that reported after 30 second cycling sprints [5], [6], [27], [28], maximal running sprints on a non-motorised treadmill [5], [19] or during isometric, isokinetic [27] or electrical stimulation [7], [20] muscle contractions. The calculated ATP production from glycolysis (64%) and from phosphagens (36%) during the whole leg press exercise agrees with the values of 60 to 70% (glycolysis) and from 30 to 40% (phosphagens) reported in maximal sprint runs in a non-motorised treadmill [19], maximal cycling [5], [18] or isometric contraction [29] exercises lasting around 30 s, assuming a metabolically closed compartment. Our finding of the increase in anaerobic glycolysis between the second and the first 5 repetitions agrees with the increased anaerobic glycolysis observed by Hultman and Sjoholm [20] during the final part of 30 s of electrical stimulation of knee extension with occluded circulation. However, other authors have found that during the last part of a 20–30 s all-out sprint cycling exercise [5], [27], [28], [30] or one-legged knee-extensor exercise at a constant work rate [4], there is a decrease in anaerobic glycolysis compared with the initial part of the exercise. This difference is likely due to differences in exercise mode (cycling, running, electrical stimulation), type of contraction (isometric-isotonic, intermittent-sustained, constant-changing work rate), frequency, intensity, duration of muscle contraction, type of muscle and species, and different amounts of lactate efflux from the active muscles.

At the end of the first 5 repetitions of leg press exercise a significant drop in power output was observed. The decrease in power output was accompanied by a parallel decrease in the concentration of PCr and energy charge as well as by an increase in ADP content in muscle whereas muscle AMP remained unchanged. Increases in the estimated muscle-free ADP content, without any changes in estimated free AMP, have been observed during 15 s sprint isokinetic cycling exercise [28]. The decrease in energy charge observed after the first 5 repetitions has been found after maximal exercise [31], indicates a diminished capacity to do work and seems to fill the purpose of energy conservation [23].

No significant changes in ATP and IMP muscle concentrations were observed during exercise despite the fact that at the end of the exercise the demand for ATP was at its highest. The finding of a lack of IMP accumulation or ATP decrease during exercise in the present study is consistent with other studies showing that no changes in muscle IMP, ATP or NH_3_ occur until PCr levels have significantly decreased to values below ∼7 mmol·Kg^−1^ wet wt [9], [22], muscle lactate reaches 12–18 mmol·Kg^−1^ wet wt [32] and muscle pH values have fallen below 6.6 [32]. The absence of changes in ATP and IMP concentrations in parallel with a decrease in power production indicates that fatigue occurred well before the metabolic conditions necessary to elicit major metabolic stress and a higher rate of AMP deamination could be established [33].

The estimated rates of glycogenolysis and glycolysis were based on increases of hexosemonophosphates and lactate. The finding of a significant increase of F-6-P during exercise suggests that the rate of glycogenolysis was higher than the rate of glycolysis. A higher rate of glycogenolysis compared to the glycolysis rate is in agreement with studies on maximal cycling [5], [27] and isometric [20] exercises lasting 30 s [20]. Furthermore, the rate of both glycogenolysis (40%) and glycolysis (50%) increased throughout the exercise in connection with decreases in PCr concentration and ATP/ADP ratio as well as with increases in ADP concentrations. The changes in glycolytic intermediates were consistent with rate-limiting steps in the phosphofructokinase, pyruvate dehydrogenase [27] and glycogen phosphorylase [34] reactions. In this hypothesis, control of the regulatory glycolytic enzymes in this manner would prevent a continued high rate of muscle glycogen utilisation and accumulation of H^+^ [26]. However, conclusions about the rate-limiting steps in these pathways, made on the basis of changes in concentrations of intermediates, should be considered to be tentative rather than final.

### Anaerobic ATP production

In agreement with previous studies that have used cycling, treadmill running or one-legged knee-extensor exercise at a constant work rate [2], [4], [6], [9], [11], [12], [18], [19], [21], [27], [30], the anaerobic energy production was estimated from muscle biopsies before and after exercise on the basis of the decrease in muscle ATP and PCr, as well as the accumulation of metabolites such a lactate. Limitations in these estimations, however, exist because it is difficult from measurements on muscle biopsy material to determine the anaerobic energy turnover during whole body exercise such as cycling, treadmill running or knee extension exercise, because the mass and the activity of the muscles involved are unknown [4]. Furthermore, the metabolic response of the biopsied muscle may not be representative of all of the muscles included in the exercise and the muscle sample may not properly interpret recruitment patterns [4]. Another problem is that the release of metabolites into the blood from the exercising muscles is frequently not taken into account when energy turnover is calculated, although this may represent a substantial contribution to the total energy production when the exercise lasts more than a few seconds [4].

Taking into account the above limitations in the estimations of the anaerobic energy production, when the muscle was regarded as a metabolically closed compartment which does not exchange metabolites with its surroundings, the calculated total anaerobic ATP production was 9% greater during the second 5 repetitions compared with the first 5. However, since the exercise was dynamic and the circulation was not restricted, some lactate diffused into the circulation during the exercise. When the amount of lactate released from the muscle to the blood during exercise was estimated from the changes in average blood lactate (see Results section) the difference in the total anaerobic energy release between the second and the first 5 repetitions increased from 9% (closed system) to 19% (adding the lactate released from the muscles). In the above calculations of ATP production during contraction the contribution from oxidative metabolism was not included. Although the aerobic metabolism was not actually measured in this study, Dudley et al. [35] have shown that during one set of 10 repetitions leading to failure of bilateral leg press exercise, pulmonary oxygen uptake increased throughout the exercise, reaching 100% higher levels during the second 5 repetitions than the first (∼50% versus ∼25% of maximal aerobic power reached during treadmill running). Moreover, it has been shown that during unilateral knee extensions oxygen utilisation of the contracting muscles is much more rapid than suggested previously and muscular and pulmonary oxygen uptake kinetics are functionally identical [10], [36]. Taken together, these findings suggest that a significant amount of ATP production during leg press exercise could come from aerobic metabolism and that aerobic ATP production was substantially greater during the second 5 repetitions than the first. The higher aerobic contribution during the second 5 repetitions would make the observed difference in ATP production between the second and the first repetitions even higher than the difference observed when only the anaerobic contribution is considered. Since the total amount of external work sustained by the quadriceps was practically the same for the first and the second 5 repetitions, the significantly higher energy cost per unit of work during the second 5 repetitions implies a reduction in mechanical efficiency, expressed as work carried out per ATP production, in the final part of exercise. This finding is in agreement with previous studies using leg extension model [4], [11].

### Why does mechanical efficiency decrease in the final part of exercise?

Our results could indicate that the efficiency of converting chemical energy into mechanical power is high in the transition from rest to leg press exercise, and then gradually declines in proportion to the source of ATP production. This is in contrast, however, to static electrical stimulation [7], [9], [37], one-legged isometric knee-extensor exercise [8], or sprint cycling exercise [30], in which decreases in the energy cost per force or per unit of work or power produced have been observed when contractions are extended over time.

Several reasons may explain the reduction in mechanical efficiency observed during the second 5 repetitions of leg press exercise. Firstly, the increased proportion of energy derived from glycolysis and oxidative phosphorylation estimated during the second 5 repetitions would increase the heat associated with ATP hydrolysis [11], [12], [37] and, therefore, would decrease the efficiency of conversion of chemical energy into mechanical power. This increase in energy derived from anaerobic glycolysis found during leg press exercise is opposite to the decrease in anaerobic glycolysis observed in the studies in which mechanical efficiency increased throughout exercise [7]–[9], [30], [37]. Secondly, the decrease in the ATP/ADP ratio observed after the first 5 repetitions can reduce the amount of energy released per mol of ATP hydrolysed, and may limit the rate of energy-requiring processes or even block energy-requiring reactions, forcing more ATP to be hydrolysed for a given amount of work [31]. Thirdly, it has been shown in mammalian muscle that during isotonic contractions at high speeds of muscle contractions, slow-twitch muscles are less efficient in doing external work than fast muscles [3]. Therefore, selective fatigue of fast-twitch fibres and progressively greater recruitment of slow-twitch fibres throughout exercise might explain the higher energy cost per unit of work observed during the final part of leg press exercise. And fourthly, the progressive increase in biceps femoris activity that has been observed during a set of 10 repetitions leading to failure of leg press exercise [38] may detract from the force produced by the quadriceps and decrease mechanical efficiency when approaching exhaustion.

### What causes fatigue during leg press exercise

The cause of muscle fatigue during maximal intense exercise remains uncertain, although many biochemical and electrophysiological changes that accompany fatigue have been described. In the present study the fall in power production during exercise was strongly correlated to the fall in phosphagens and the increase in muscle lactate. Associations between decline in muscle tension and proportional changes in muscle phosphagens, as well as changes in muscle lactate or pH have been observed in many studies during repeated isometric contractions in frog stimulated muscle using ^31^P NMR [39] and during maximal short-term dynamic exercise in humans [40]. Thus, it seems reasonable to suggest that some biochemical changes, such as a decrease in the contribution of PCr [18], an abrupt increase in muscle lactate and by-products of ATP hydrolysis (H^+^, P_i_, ADP) and the inability of the glyconeogenolytic rate to compensate for the fall in ATP production when the PCr store is depleted [18], [26], [29] contributed to fatigue during leg press exercise.

In agreement with our results, decreases in the ATP production rate over time have been observed during isometric contractions [29] lasting around 30 s and during the second half of a 30-s maximal sprint-cycling exercise [28]. The calculated anaerobic ATP turnover rate of 1.09 mmol·Kg^−1^ wet muscle·s^−1^ observed during this whole exercise (10 repetitions), corresponds well with earlier estimates for human muscle where values ranging from 0.5 to 2.0 mmol·Kg^−1^ wet wt·s^−1^ were reported in cycling [5], [6], [18], [27], [28] and maximal running sprints [19] or during isokinetic or electrical stimulation [20], [41] exercises lasting 10–30 s. These values of anaerobic ATP turnover rate are, however, lower than the highest rates for PCr and glycolytic ATP provision reported during all-out 6 s sprints [21]. The decrease in the energy rate of utilisation as the exercise progresses has been related to a decline in the rate of ATP production as a result of PCr hydrolysis [29], the accumulation of ADP slowing down cross-bridge ATP utilisation [42], phosphorylation of the so-called regulatory light chain of myosin, regulation by intracellular acidosis, accumulation of inorganic phosphate (P_i_) or lowered ATP/ADP ratios [43], [44]. Our results are consistent with the concept that the decrease in power output during leg press exercise is caused by the reduction in the capacity to generate ATP at a high rate from anaerobic sources [45].

In summary, this data shows that during the first 5 repetitions of a set of 10 repetitions leading to failure of bilateral leg press exercise ATP is resynthesized predominantly from PCr with a smaller contribution of glycolysis, while during the second 5 repetitions this pattern is inverted, i.e. the anaerobic ATP resynthesis is produced mostly by glycolysis. Over the repetitions power output is reduced and the work is produced by slowing the speed of muscle contraction. This reduces the energy demand but not enough to balance ATP production and utilisation, due to the impairment of mechanical efficiency with fatigue. The mismatch between ATP production and demand, reflected by the reduction in the ATP/ADP ratio, combined with the accumulation of by-products of the energy metabolism may lead to a final task failure. It remains to be explained why mechanical efficiency is reduced during leg press fatiguing exercise.

### Application statement

Although the leg press exercise is one of the most common exercises performed by trained athletes or knee injured people, there is a significant gap in the body of knowledge pertaining to the metabolic and power output changes during bilateral leg press exercise, as no measurements have been made, and it is inappropriate to suppose that it will be the same as that found with other muscle contraction models. This study reports on the acute muscle metabolic response to a single set of this exercise. The results provide the first evidence that performing 5 or 10 repetitions of leg press exercise with the maximal load possible to achieve 10 repetitions (10RM) gives two main different types of exercise in terms of energy status and muscle metabolism: 1) the first 5 repetitions (duration ∼14 s) result in modest increases in blood (∼1.4 mmol•l^−1^) and muscle (∼5 mmol•Kg^−1^ wet muscle) lactate and in high decreases of the muscle PC (∼42%) content while muscle generating capacity are maintained, and 2) compared with the first, the second 5 repetitions (duration ∼18 s) are characterized by markedly higher increases in blood (∼2.0 mmol•l^−1^) and muscle (∼10 mmol•Kg^−1^ wet muscle) lactate that may reach average values up to 17 mmol·l^−1^ at the end, and by markedly lower decreases in muscle PC (∼19%) content leading to a significant decrease in muscle generating capacity and mechanical efficiency. Compared to the final part of leg press exercise, the lower lactate accumulation and the maintenance of muscle generating capacity during the initial part of exercise are opposite to those reported during 20–30 s all-out sprint cycling exercise or one-legged knee-extensor exercise at a constant work rate. This type of fatigue may relate to decreased contribution of PCr and/or abrupt increases in muscle lactate or by-products of ATP hydrolysis. The strong correlations found in the present study between blood and muscle lactate, as well as between changes in power output and changes in muscle lactate, indicate that blood lactate concentration may give valuable information about the changes taking place in muscle lactate and in power output during a set of leg press exercise. The present description of the metabolic response to a single set of bilateral leg press exercise is of importance in the search for a better understanding of the factors that limit performance in this type of exercise and would help to compose a scientifically-based and well-balanced training program that improves knee extension performance in trained athletes or knee injured people.
